# The influence of mTOR inhibitors on immunity and the relationship to post-transplant malignancy

**DOI:** 10.1186/2047-1440-2-S1-S2

**Published:** 2013-11-20

**Authors:** Edward K Geissler

**Affiliations:** 1 Department of Surgery, Experimental Surgery, University Hospital Regensburg, Franz-Josef-Strauss-Allee 11, Regensburg 93053, Germany

**Keywords:** mammalian target of rapamycin, post-transplant malignancy, T cells, B cells, dendritic cells, organ transplantation

## Abstract

The known role of mammalian target of rapamycin (mTOR) in the immune response has been rapidly evolving, from what was once thought to be a simple immunosuppressive antiproliferative effect on T cells to a very complex central role that serves to integrate multiple signals given to T cells, B cells and antigen-presenting cells. The complexity of this topic is demonstrated by recent data suggesting that mTOR inhibition can either inhibit or promote certain aspects of immune responses, depending on the nature of the antigenic stimulus, and the environmental conditions cueing the cellular immunological players. There is even evidence that, under mTOR inhibition, an immune response to one foreign entity (for example, an organ transplant) may be simultaneously completely different to that of another (for example, tumour or microorganism). To understand how this might be possible, it is necessary to investigate the central role that mTOR seems to have in shaping the immune response. This review is aimed at examining how mTOR controls the development and function of key immune cells, and puts this information primarily in the context of organ transplant rejection and post-transplant malignancy.

## Immunity, immunosuppression and post-transplant malignancy

A relationship between the use of general immunosuppressive drugs to prevent allograft rejection and the development of cancer after organ transplantation has been recognised for decades. The scientific transplant community has developed a growing concern about cancer, since post-transplant malignancy has emerged as a leading cause of morbidity and mortality, especially in patients who have a high or long-term exposure to immunosuppression [1,2]. There are different explanations for why post-transplant malignancy occurs more frequently in this pharmacologically immunosuppressed population, including enhancement of tumour invasive properties [3] and reduction in DNA repair mechanisms [4]. However, the most discussed mechanism is the seemingly obvious effect of suppressing the ability of immune cells to detect and eliminate cancer as it develops (immune surveillance). Although one might be intuitive to hypothesise that all immunosuppressive drugs will have the same suppressive effect against tumour immunity, recent research suggests that this may not be the case. New basic questions have therefore been raised, including the following: how do mammalian target of rapamycin (mTOR) inhibitors affect the development of specific immune cells that are most critical to produce an effective anti-tumour immune response? Do the various immunosuppressive drugs affect these cells differently? Is it possible to enhance an immune response towards a tumour, while at the same time inhibiting immunity towards a transplanted allograft? Since the most common tumours that develop in transplant recipients are virally associated [5], how do mTOR inhibitors influence specific viral infections in these patients?

Certainly intriguing is the fact that specific immune responses formed simultaneously against an allograft and a (immunogenic) tumour may be different in nature, and are likely to be altered significantly by various immunosuppressive substances, depending on the answers to the questions posed above. The present review focuses on new evidence that the mTOR pathway is uniquely positioned to affect the differential development of lymphocyte subpopulations, as well as the maturation of antigen-presenting cell (APC) populations, all of which are critical in the formation of immune responses towards organ transplants and tumours. Interestingly, some of the affected immune cell types sway immunity towards suppressive regulation, and others enhance effector cell capabilities. With regard to organ transplantation recipients, the ideal overall effect sought therapeutically is complex, and demanding, in that it is desirable to minimise the immune response against the allograft, while at the same time enhancing immunity against tumours (and infectious microorganisms). Although this suggestion appears to be mutually exclusive, there are reasons to believe that the development of such a diametrically opposing response to an organ transplant and a tumour or microorganism may be possible. In this article the effects of mTOR inhibitors on different cellular components of the immune system are reviewed. The aim is to put forward the latest information regarding the effects of mTOR inhibition on immunity in the context of reducing the complex problem of post-transplant malignancy in transplant recipients.

## Immunosuppressive and immunomodulatory effects of mTOR inhibitors

Inhibitors of mTOR have been used for more than a decade as immunosuppressive agents to prevent organ transplant rejection. Early mechanistic studies on rapamycin pointed towards a rather simple explanation for the immunosuppressive effect, in that the mTOR pathway was found to be essential for the cell proliferation signal (often referred to as signal 3) triggered by IL-2, thus preventing the expansion of donor-specific T cells activated through the T-cell receptor [6]. This immunosuppressive effect contrasts in general with calcineurin inhibitors (CNIs), which act by preventing the initial activation (signal 1) of T cells [7]. While our overall mechanistic understanding of the basic suppressive activity of CNIs is still generally accepted, new data suggest that the suppressive effect of mTOR inhibition on immune cells is very complex and probably does not rest primarily on inhibition of T-cell proliferation.

**Figure 1:**
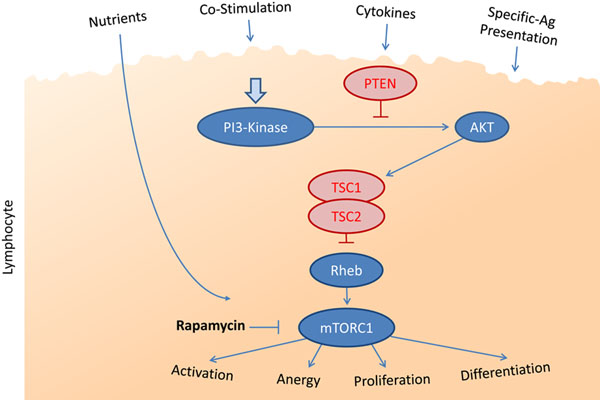
**Integration of various signals through mammalian target of rapamycin in lymphocytes.** PTEN, phosphatase and tensin homolog; Rheb, Ras homolog enriched in brain; TSC, tuberous sclerosis.

Recent mechanistic investigations indicate that mTOR plays a central role in the differentiation of T-cell subsets, and also controls aspects of B-cell and APC development. In fact, mTOR is a critical regulator of the immune response because it acts as a central node for sensing nutrient availability, cytokine/growth factor signalling and co-stimulatory factors (Figure 1). Indeed, mTOR is in a unique intracellular signalling position to integrate all of these factors so cells can effectively and properly balance cues from the ever-changing microenvironment, such as those induced by microbiological (for example, bacterial, viral) or allogeneic (for example, organ transplant, tumour) challenges.

## Role of mTOR in immune cell development – T cells

T cells are critically involved at nearly all levels of any immune response. While the primary effect of mTOR inhibition on T cells was initially attributed to blockage of IL-2 proliferation-inducing signalling, hints that this is not the only effect have become evident. One clue was that the initial finding that rapamycin treatment induces T-cell anergy (lack of responsiveness) through inhibition of proliferation [8,9] was later found to be independent of this anti-proliferative effect, and rather to be due to a direct inhibitory effect on mTOR itself [10]. Subsequent investigations into the link of mTOR to T-cell metabolism, and to transcription factors that are now recognised to control T-cell subset differentiation, opened new views towards mTOR inhibitor effects on the immune response.

Regarding metabolism, mTOR’s central role comes directly into play because activated lymphocytes primarily use glycolysis for energy due to their need to produce proteins, nucleotides and lipids that are essential for the generation of key biosynthetic substrates [11,12]; the shifting from mitochondrial respiration to glycolysis (referred to as the Warburg effect) is similar to that which occurs in cancer cells. Interestingly, mTOR as a regulator of metabolism provides links to lymphocyte activation in this context. One example is that T-cell co-stimulation via CD28 triggers the activation of signalling molecules upstream of mTOR that promote expression of necessary membrane glucose transporters. In general, one can state that inhibition of cell metabolism through mTOR leads to inhibition of T-cell-mediated immunity. The importance of this idea cannot be overstressed since it has been shown, for instance, that T-cell anergy is due at least in part to decreased mTOR activation [13]; if mTOR is resistant to reactivation in an anergic state, then the required metabolic machinery is not going to be available and the cell will remain anergic to otherwise stimulatory signals. Indeed, substances such as metformin and AICAR, which mimic energy depletion and activate AMPK (an inhibitor of mTOR), promote T-cell anergy [13-15]. Cell metabolism, mTOR and the immune response currently constitute an intense area of basic research that has substantial therapeutic potential and implications.

**Figure 2:**
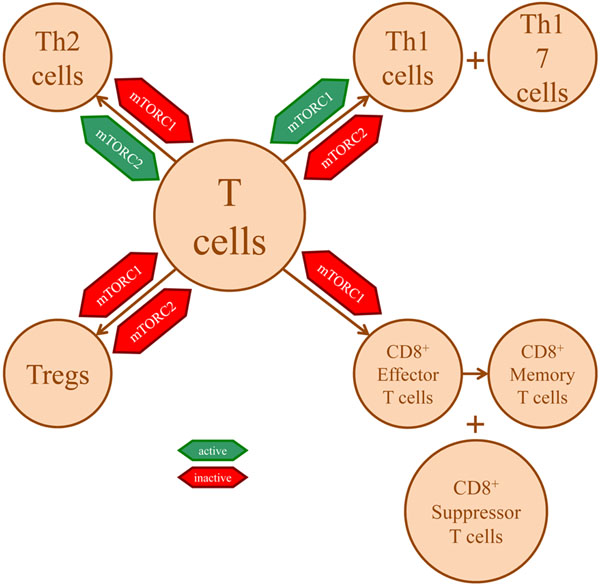
**Selective mammalian target of rapamycin complex 1/2 blockade leads to differential development of T-cell subpopulations.** Selective blockade of mammalian target of rapamycin complex 1 (mTORC1) and mammalian target of rapamycin complex 2 (mTORC2) leads to differential development of T-cell subpopulations. Tregs, T-regulatory cells.

The differentiation of CD4 and CD8 T-cell populations has a major impact on the development of any immune response to allogeneic transplants or tumour entities. Recent data demonstrate an important role for mTOR in determining the T-cell differentiation pattern (Figure 2). To understand this role better, first it is necessary to recognise that mTOR is part of two large complexes, referred to as mTOR complex 1 (mTORC1) and mTOR complex 2 (mTORC2), where mTORC1 is directly inhibited by rapamycin while mTORC2 is only indirectly and partially inhibited with long-term exposure to the drug. Second, it is helpful to know that differentiation of Th1, Th2 and Th17 T-helper cell subsets is regulated by the lineage-specific transcription factors T-bet, GATA-3 and RORγt, respectively. Considering this background information, recent experimental models suggest that blocking of mTORC1 with rapamycin, or by knocking out essential components of the mTORC1, a Th2 polarised T-cell dominance develops; whereas knocking out mTORC2 polarises the T-helper immune response towards Th1 and Th17 cell development [16]. Most interestingly, blocking both mTOR complexes leads to the generation of a Foxp3^+^ T-regulatory (Treg) cell expansion. Moreover, these Treg cells are resistant to apoptosis [17]. Indeed, Treg cells appear in general to require less mTOR activity, which is consistent with the reduced metabolic demands for these cells compared with effector T cells [18]. Interestingly, although Treg cells depend on IL-2 for proliferation, IL-2 stimulation results in high levels of STAT5 phosphorylation, rather than activation of mTOR [19], suggesting that different T-cell subpopulations depend on alternate signalling pathways for expansion and survival. In terms of therapeutic application of mTOR inhibitors, research suggests that the differential effects summarised above depend substantially on the dose, duration and timing of the drug application [20-22], indicating that more is to be learnt about how best to apply mTOR inhibitors to suit the clinical purpose intended.

Some of the same effects apply to CD8^+^ cells regarding mTOR dependence. For instance, activation of CD8^+^ cells also primarily depends on glycolysis [23], and differentiation of effector CD8^+^ cells requires mTORC1-dependent T-bet expression [24]. Most critically, mTOR is involved in the transition of effector to memory CD8^+^ T cells (Figure 2), and this appears to rely on conversion of T-bet to eomesodermin transcription factor expression [24-26]; blocking mTOR with rapamycin has this exact effect, and therefore promotes the development and sustenance of memory T cells that transition efficiently into effector cells highly capable of producing immune responses to, for instance, tumours [24]. Similar to Treg cells, memory CD8^+^ T cells depend on mitochondrial oxidative phosphorylation for energy (rather than glycolysis) and are driven by STAT5 signalling. One perplexing question is therefore whether mTOR inhibition increases immunity to viruses, bacteria and tumours, while at the same time protects organ transplants from rejection.

Recent data suggest that rapamycin treatment augments CD8^+^ T-cell memory responses towards viruses. This effect has been demonstrated by impressive boosting of vaccination responses both in mice [27,28] and in non-human primate studies [29]; in the nonhuman primate experiments, immunosuppressive doses of sirolimus promoted CD8^+^ T-cell memory towards vaccinia virus, while CNI use did not. Indeed, it is ironic that an immunosuppressive agent is being considered for boosting vaccination responses in humans. Another interesting aspect of this research is that viral infections (for example, human papillomavirus, hepatitis B/C, Epstein–Barr virus, human herpesvirus 8) are associated with the most common post-transplant malignancies, suggesting that a boost in immunity to these viruses could affect cancer development. Moreover, several recent experimental studies indicate that rapamycin administration directly enhances memory T-cell formation against tumours [22,24]. This is an observation we have also been able to confirm in the laboratory [30], and we can add that CNIs do not support memory development in our models. The boosting of T-cell memory with mTOR inhibition has substantial therapeutic implications regarding the problems of viral infection and post-transplant malignancy in organ transplant recipients.

This leads to the question raised earlier of whether an immune response can be promoted in one foreign entity (for example, virus, bacteria or tumour cell) and yet inhibited by another (an organ transplant). An interesting experimental study from Ferrer and colleagues demonstrates that rapamycin-treated mice have protection against rejection of an OVA-expressing skin allograft, while at the same time showing a heightened CD8^+^ T-cell response against the same OVA epitope expressed by bacteria (*Listeria monocytogenes*) [31]. This is a critical observation, since it opens the possibility that mTOR inhibitors can enhance immunity to infectious agents without at the same time promoting the immune reaction against an organ allograft. In fact, it can be argued that enhancement of CD4^+^ Treg cell and CD8^+^ T suppressor cell [32] responses towards allografts may provide for long-lasting protection and perhaps even some degree of immunological tolerance. Unfortunately, it is completely unclear why there is such a divergent response to two foreign entities expressing the same foreign protein. Does this divergence relate to the microenvironmental conditions under which allograft versus microbiological antigens are presented to the immune system, or are other factors responsible? This is clearly an intriguing area of research, and highlights the importance of mTOR’s role in orchestrating complex immune responses.

## Role of mTOR in B-cell and antigen-presenting cell development

Less information exists regarding the role of mTOR inhibitors on B cells. However, data from the mTOR hypomorph mouse (constitutive reduced, but not absent, mTOR levels) suggest that B-cell development may be even more affected than T cells [33]. In these mice, B-cell development in the bone marrow is partially inhibited, which was reflected by decreased B-cell proliferation in response to antigenic stimulation and reduced antibody production capability. Interestingly, mice with B cells that overexpress mTOR because of a TSC1 deletion (TSC1 normally inhibits mTOR, see Figure 1) also demonstrate similar defects in B-cell differentiation and antibody production [34]. Another indication for an mTOR role comes from the fact that activated B cells, like T cells, use glycolysis as a primary source of energy [35]. Together, these early experimental indications suggest that mTOR is likely to have a significant impact on B-cell activation, differentiation and function, but more in-depth studies are lacking to define the exact role of mTOR in B-cell-mediated immunity.

Finally, the mTOR pathway is also important for the differentiation and function of APCs. In particular, mTOR inhibition has a potent effect on the maturation of dendritic cells (DCs). Differentiation into conventional, CD8^+^ and plasmacytoid DCs appears to depend on mTOR. Indeed, mice with uninhibited mTOR activity (via PTEN deletion, see Figure 1) develop an abnormal highly expanded DC compartment [36], suggesting that mTOR plays a critical role in maintaining APC homeostasis *in vivo*. Moreover, rapamycin treatment has a profound effect on APC function, in that co-stimulatory molecule expression is decreased, leading to an inhibited ability for APC to stimulate T-cell activation [37,38]. Rapamycin-treated DCs are even known to induce tolerance in animal models [39,40], possibly through their ability to promote the development of Treg cells [41]. In fact, researchers that are expanding Treg cells for the purpose of cell therapy often use rapamycin to produce a more stable Treg phenotype [42]. One should mention, however, that rapamycin has seemingly opposing effects on the early development of immune responses involving plasmacytoid DCs versus other DCs. While plasmacytoid DCs activated via toll-like receptors depend on mTOR to elicit type 1 interferon-based expression responses, lipopolysaccharide activation of monocytes and DCs leads to inhibition of a proinflammatory gene expression pattern through the mTOR pathway [43]; mTOR inhibition could thus affect responses to bacterial challenges, especially in immunosuppressed transplant recipients. mTOR therefore has important effects on APC homeostasis and development that require a great deal more research to be fully understood. Nonetheless, mTOR clearly has yet another key function in the development of immune responses.

## Conclusion

What was once thought to be a simple explanation (antiproliferative effect) for how mTOR inhibitors reduce the immune reaction to organ allografts is now developing into a very complex explanation. One should also state that while several nonimmunological mechanistic explanations for the anti-tumour effects of mTOR inhibitors have been described [44], promotion of immune responses to cancer is unexpectedly coming more into focus. The most recent data suggest that mTOR acts as a central node for coordinating activities of the most important cells (T cells, B cells and APCs) forming the immune response to various challenges. Interestingly, some of these effects inhibit an immune response, and other effects actually promote immunity; the setting of the antigenic challenge appears to be crucial, since energy availability, signalling cues and cell activation all converge to at least some degree upon mTOR.

What does this mean for transplant patients in terms of allograft protection (immunosuppression), viral or bacterial infection and post-transplant malignancy? Although there are no simple answers to this question, more light is being shed on the topic with intensive ongoing research. In terms of protecting allografts from rejection, mTOR inhibitors are attractive from the theoretical perspective that they might be optimal for maintaining a state of donor-specific regulation through promotion of tolerogenic DCs and Treg cells. Although mTOR inhibitors alone do not appear to produce tolerance in transplant recipients [45,46], perhaps strategic use of these drugs in combination with novel induction therapies or cell therapy [47,48] could yield better results. Regarding infectious complications associated with immunosuppression in organ transplantation, there is already early evidence that mTOR inhibitors might reduce the problem of some viral infections, including cytomegalovirus, human herpesvirus 8 and BK virus [49-54]. In turn, if viral infections can be decreased, mTOR inhibitors could have an indirect impact on the development of post-transplant malignancies. In addition, promotion of memory CD8^+^ T-cell responses against tumour cells could also lessen the problem of cancer in transplant recipients.

To conclude, although early evidence suggests that mTOR inhibitors have the potential to promote an immune response against an infectious microorganism or tumour entity, and can paradoxically function to inhibit immunity against an organ allograft, further research is needed to untangle the operative mechanisms and to ultimately explore the full potential of mTOR inhibitors in the setting of organ transplantation.

## Abbreviations

APC: antigen-presenting cell; CNI: calcineurin inhibitor; DC: dendritic cell; IL: interleukin; mTOR: mammalian target of rapamycin; mTORC1: mammalian target of rapamycin complex 1; mTORC2: mammalian target of rapamycin complex 2; Th: T-helper; Treg: T-regulatory.

## Competing interests

The author declares that he has no competing interests.

## Declaration

This article has been published as part of *Transplantation Research* Volume 2 Suppl 1, 2013: Proceedings of the 12th International Symposium on Transplantation. The full contents of the supplement are available at http://www.transplantationresearch.com/supplements/2/S1. The supplement is based on the 12th International Transplantation Symposium held on 16–17 April 2012, in Istanbul, Turkey. The symposium and publication based on these proceedings were supported by an unrestricted educational grant from Pfizer Inc., who had no influence on the editorial content. Editorial support was provided by Synergy, Richmond, UK and was funded by Pfizer Inc. The articles have been through the journal’s standard peer review process and the Supplement Editors declare that they have no competing interests.
